# Knowledge mapping of Cushing syndrome: A bibliometric analysis from 2000 to 2023

**DOI:** 10.1097/MD.0000000000046025

**Published:** 2025-11-21

**Authors:** Xiao Lu, Mingyu Huang, Yufang Yang, Dongxiao Chen, Fuli Qin, Quanyuan Huang

**Affiliations:** a Department of Pharmacy, The First Affiliated Hospital of Guangxi Medical University, Nanning, Guangxi Zhuang Autonomous Region, China; b Guangxi Medical University, Nanning, Guangxi Zhuang Autonomous Region, China; c Affiliated Hospital of Yulin City Health School, Yulin, Guangxi Zhuang Autonomous Region, China.

**Keywords:** 11β-hydroxylase inhibitor, bibliometric analysis, Citespace, Cushing syndrome, somatic mutation, VOSviewer

## Abstract

**Background:**

Cushing syndrome (CS) is an endocrine disorder primarily caused by cortisol overproduction, resulting in a range of clinical manifestations. Although recent studies have advanced the understanding and treatment of CS, no comprehensive bibliometric analysis has yet mapped the development and trends in this field. This study aimed to provide a detailed overview of the knowledge landscape, research output, and emerging hotspots in CS research using bibliometric methods.

**Methods:**

A bibliometric analysis was conducted using publications on CS indexed in the Web of Science Core Collection from January 2000 to October 2023. VOSviewers, CiteSpace, and the R package bibliometrix were used to analyze publication outputs, author collaborations, institutional and country-level contributions, journal impact, co-citation networks, and keyword trends.

**Results:**

The volume of CS-related publications has shown a steady annual increase. A total of 4661 articles were published across 86 countries. The United States produced the highest number of publications, while the Eunice Kennedy Shriver National Institute of Child Health and Human Development ranked as the most active institution. The *Journal of Clinical Endocrinology and Metabolism* was the leading source both in terms of publication count and co-cited frequency. The 19,621 contributing authors included several high-output researchers, notably Constantine A Stratakis, Martin Reincke, Jerome Bertherat, Susan M. Webb, and Felix Beuschlein. Keywords analysis identified “11β-hydroxylase inhibitors” and “somatic mutation” as prominent emerging topics.

**Conclusion:**

This bibliometric study presents the first comprehensive mapping of global CS research over the past 2 decades. It identifies influential contributors, collaborative networks, and thematic developments, offering a structured reference for future research planning and scholarly engagement in the field of CS.

## 1. Introduction

Cushing syndrome (CS) is a clinical condition caused by prolonged exposure to elevated levels of cortisol. It may result from the use of exogenous glucocorticoids, endogenous overproduction of adrenocorticotropic hormone by the pituitary gland or ectopic sources, or autonomous cortisol secretion by adrenal tumors.^[1]^ Typical clinical manifestations include centripetal obesity, facial rounding (“moon face”), dorsocervical fat pad (“buffalo hump”), and thinning of the skin. Patients are also at increased risk of serious complications, including hypertension, metabolic disorders, osteoporosis, and impaired immune function.^[1]^ Early diagnosis and treatment are essential for reducing morbidity and mortality, as well as for mitigating long-term complications associated with hypercortisolism.^[2,3]^ The growing body of literature on CS reflects the complexity of its pathophysiology, diagnosis, and management, and offers important evidence to guide clinical practice.

Bibliometrics is a quantitative method for evaluating scientific literature and understanding research dynamics within a specific field.^[4]^ It employs statistical techniques to assess indicators such as publication volume, citation frequency, author contributions, collaboration networks, and journal impact.^[4]^ Bibliometric analysis provides a structured framework for exploring scientific output, identifying knowledge gaps, and informing research priorities and policy decisions.^[5]^ Widely used tools for such analysis include VOSviewer and CiteSpace, which enable visual mapping of citation networks, keyword co-occurrence, and author collaborations. These tools, along with the Web of Science Core Collection database, form the methodological basis of this study.^[6]^

This objective of this study is to systematically map the knowledge base and evolving research trends in CS through bibliometric analysis. By examining global publication patterns from 2000 to 2023, we aim to identify influential authors, leading institutions, core journals, collaborative networks, and emerging research themes. This analysis offers a consolidated overview of the field of CS research, supporting scholars and clinicians in understanding the current landscape and guiding future research directions.

## 2. Materials and methods

### 2.1. Literature search and data collection

**Figure 1.**
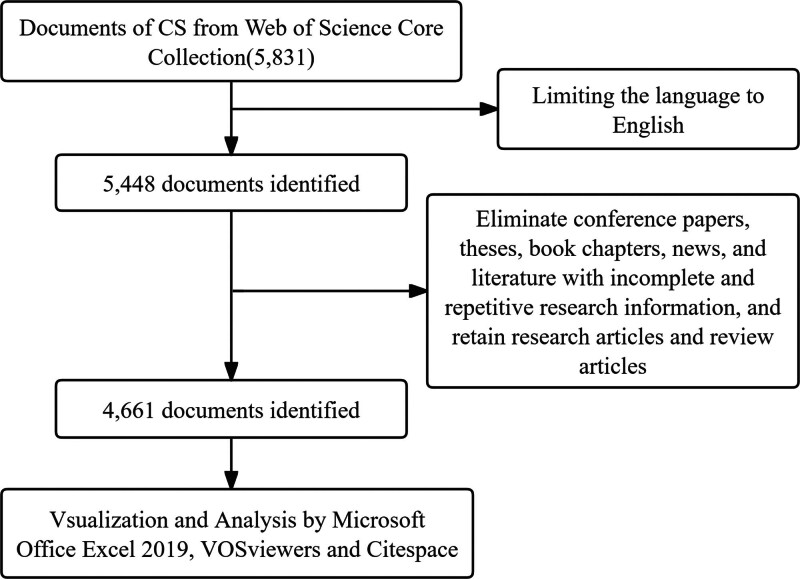
Methodological flowchart of the study.

A literature research was conducted on November 11, 2023, using the Web of Science Core Collection database. The search strategy was as follows: TS = (“Cushing syndrome”) OR TS = (“Cushing syndrome”). The search period was restricted to publications from January 2000 to October 2023. A total of 5831 records were initially retrieved. After limiting the language to English, 5448 publications remained. Conference proceedings, theses, book chapters, news articles, and records with incomplete or duplicated information were excluded. Only original research articles and review articles were retained, generating 4661 records for bibliometric analysis (Fig. 1). This study did not involve human participants and animal subjects and therefore did not require institutional ethical approval.

### 2.2. Bibliometric and statistical analysis

Core bibliometric methods were applied in this study. These included: co-occurrence analysis, used to identify relationships among entities such as keywords, countries, and institutions; co-citation analysis, to examine the intellectual structure of the field; citation burst detection, based on Kleinberg algorithm, to identify emerging research trends; timeline visualization, to trace thematic evolution using average publication year; and cluster analysis, to classify network nodes into coherent research sub-domains.

Primary analyses were performed using VOSviewer (version 1.6.19; Leiden University, Leiden, The Netherlands), including country and institution mapping, journal and co-cited journal analysis, author and co-cited author mapping, reference co-citation analysis, and keyword clustering to identify hotspots and research frontiers. In visualizations generated by VOSviewer, nodes represent entities such as countries, institutions, journals, or authors. Node size and color denote quantity and category, respectively, while line thickness reflects the strength of collaboration or co-citation relationships.

CiteSpace software (version 6.1.R6; Drexel University, Philadelphia) was employed for journal dual-map overlays, and citation burst detection of references and keywords. Bibliometrix, an R-based package (version 3.2.1; https://www.bibliometrix.org), was used for trend and performance analysis. Journal quartile rankings and impact factors were sourced from *Journal Citation Reports 2023*. Descriptive statistics were performed using Microsoft Excel 2019 (Microsoft Corporation, Redmond).^[7,8]^

## 3. Results

### 3.1. Overview of publication details

**Figure 2.**
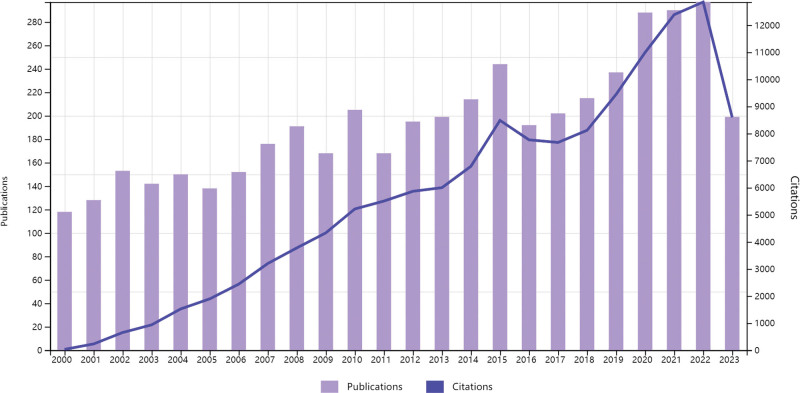
Annual publication trends in CS research from 2000 to 2023. CS = Cushing syndrome.

The annual number of publications reflects the pace and direction of research in a given field. As shown in Figure 2, from 2000 to 2009, the number of publications on CS exhibited a gradual upward trend, ranging from 110 to 200 articles per year. Between 2010 and 2019, the annual output stabilized, fluctuating around 200 articles. From January 2020 to October 2023, annual publications exceeded 280. Overall, both the number of publications and total citations have shown a consistent increase, indicating growing scholarly attention and sustained research activity in this field.

### 3.2. Analysis of countries and institutions

A total of 4661 papers on CS were published by 3972 institutions across 86 countries. The 10 most productive countries and institutions are listed in Table 1. The United States ranked first, contributing 1205 publications and receiving 52,833 citations. Notably, 6 of the top 10 institutions are based in the United States and Italy, highlighting these countries’ central role in CS research. Leading institutions, such as the Eunice Kennedy Shriver National Institute of Child Health and Human Development and the University of Padua, are frequently involved in collaborative research initiatives, both domestically and internationally.

**Table 1:** Top 10 countries and institutions contributing to CS-related scholarly works.

Rank	Country	Documents	Citations	Rank	Organization	Documents	Citations
1	USA	1205	52,833	1	Eunice Kennedy Shriver National Institute of Child Health and Human Development	118	5681
2	Italy	575	24,441	2	University of Padua	113	5882
3	Japan	422	6855	3	University of Milan	87	3454
4	England	391	21,372	4	Mayo Clinic	86	6459
5	Germany	348	13,573	5	National Institute of Child Health and Human Development	84	8050
6	France	315	16,619	6	University of Turin	76	6308
7	China	292	2743	7	Leiden University	70	3752
8	Netherlands	254	12,004	8	University of Birmingham	67	6372
9	Spain	181	4310	9	Erasmus University Rotterdam	64	3974
10	Canada	165	9687	10	University of Paris Descartes	63	5766

CS = Cushing syndrome.

**Figure 3.**
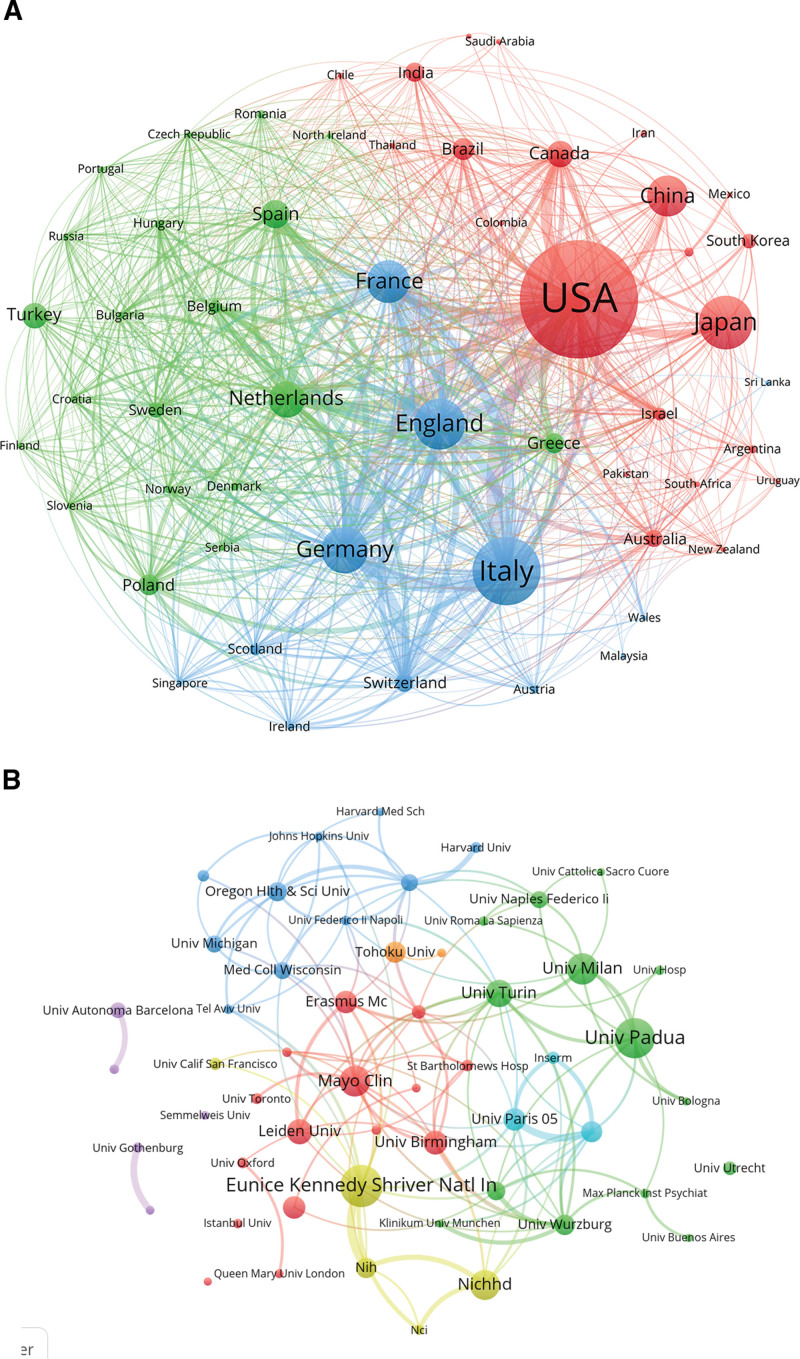
(A) Country co-authorship visualization map based on CS publications. (B) Institutional co-authorship visualization map in the field of CS. CS = Cushing syndrome.

A co-occurrence network was generated using countries with at least 5 publications and institutions with at least 24 publications (Fig. 3A and B). The network maps show that the United States, England, Italy, Japan, and France are represented by larger nodes and thicker connecting lines, indicating higher publication output and stronger collaborative ties. The Eunice Kennedy Shriver National Institute of Child Health and Human Development, based in the United States, produced the highest number of publications and citations across all institutions. The international co-authorship patterns reveal that researchers from these leading countries frequently collaborate, sharing expertise, data, and resources to advance the field.

### 3.3. Analysis of journals and co-cited journals

Research on CS was published across 1021 journals. The *Journal of Clinical Endocrinology & Metabolism* was the most relevant journal, publishing 321 articles (31.44% of total publications), followed by the *European Journal of Endocrinology* with 234 articles (22.92%). *Clinical Endocrinology* and *Endocrine* also contributed significantly, publishing 155 and 129 papers, respectively. Among the top 10 journals (Table 2), the *Journal of Clinical Endocrinology & Metabolism* and *European Journal of Endocrinology* held the highest impact factors (IF = 5.8). In terms of quartile rankings, 5 journals (50%) were classified as quartile 1 (Q1), 2 (20%) as quartile 2, 1 (10%) as quartile 3, and 2 (20%) as quartile 4, indicating that the majority of CS-related research is disseminated in high-impact journals.

**Table 2:** Top 10 journals and their corresponding co-cited counterparts in the domain of CS research.

Rank	Journal	Count	IF	Q	Co-cited Journal	Co-citation	IF	Q
1	Journal of Clinical Endocrinology & Metabolism	321 (31.44%)	5.80	Q1	Journal of Clinical Endocrinology & Metabolism	29,096	5.80	Q1
2	European Journal of Endocrinology	234 (22.92%)	5.80	Q1	Clinical Endocrinology	8850	3.20	Q3
3	Clinical Endocrinology	155 (15.18%)	3.20	Q3	European Journal of Endocrinology	8635	5.80	Q1
4	Endocrine Journal	129 (12.63%)	2.00	Q4	New England Journal of Medicine	4345	158.50	Q1
5	Endocrine	122 (11.95%)	3.70	Q3	Lancet	2316	168.90	Q1
6	Frontiers in Endocrinology	116 (11.36%)	5.20	Q1	Endocrinology	2273	4.80	Q2
7	Pituitary	115 (11.26%)	3.80	Q2	Endocrine Reviews	2224	20.30	Q1
8	Journal of Endocrinological Investigation	109 (10.68%)	5.40	Q1	Journal of Endocrinological Investigation	2211	5.40	Q1
9	Hormone and Metabolic Research	61 (5.97%)	2.20	Q4	Journal of Endocrinological Investigation	2207	5.40	Q1
10	Internal Medicine	58 (5.68)	1.20	Q4	Endocrinology and Metabolism Clinics of North America	1826	4.50	Q2

CS = Cushing syndrome, Q1 = quartile 1, Q2 = quartile 2, Q3 = quartile 3, Q4 = quartile 4.

**Figure 4.**
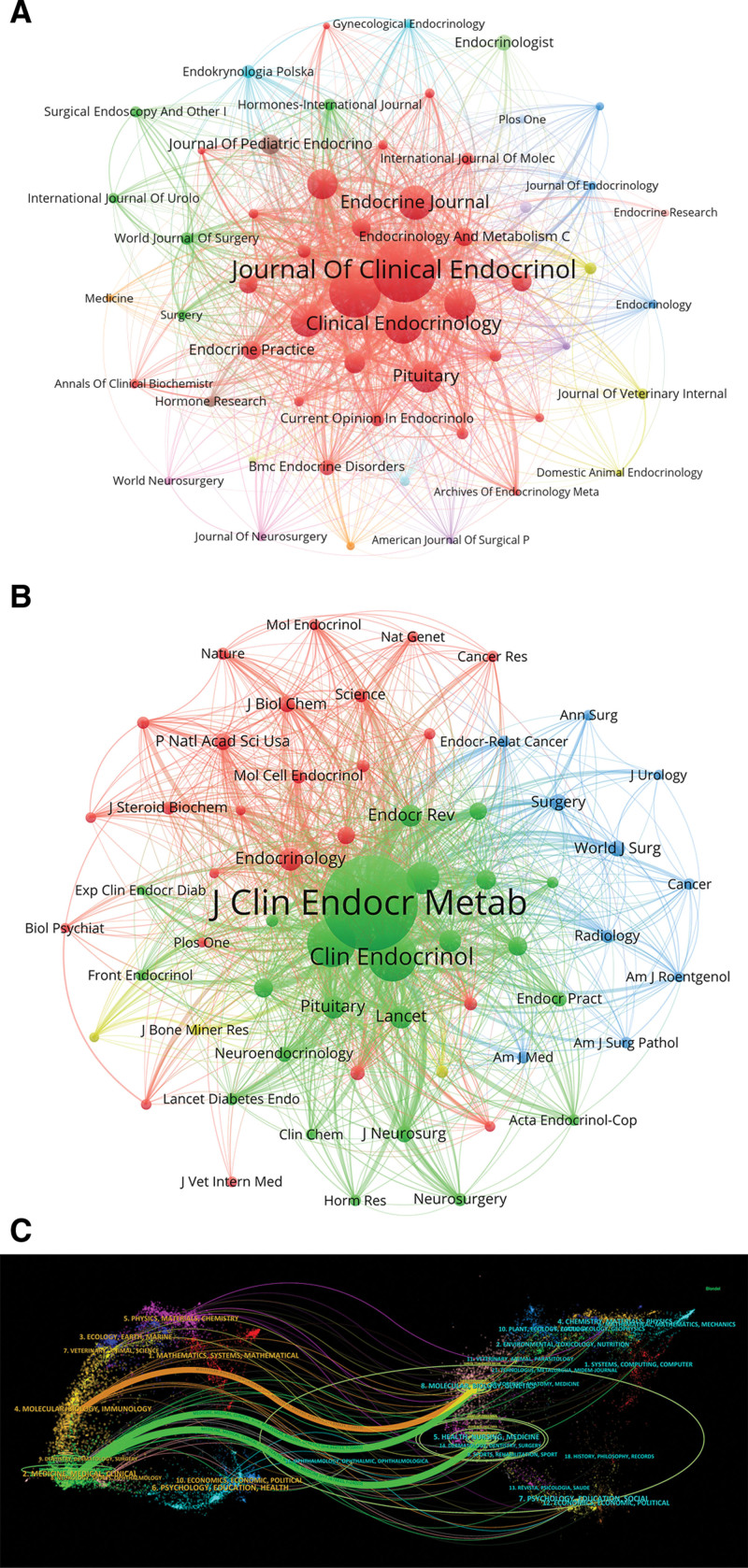
(A) Visualization of active journals publishing on CS. (B) Co-cited journals visualization map. (C) Dual-map overlayof journal citations, indicating citation paths across disciplines. CS = Cushing syndrome.

A co-authorship threshold of at least 13 publications was applied, leading to 56 journals used to construct the journal co-occurrence network (Fig. 4A). The map reveals strong citation relationships between the *Journal of Clinical Endocrinology & Metabolism* and journals such as *Clinical Endocrinology*, *Frontiers in Endocrinology*, and *Endocrinology*.

Co-citation analysis (Table 2) showed the *Journal of Clinical Endocrinology & Metabolism* ranks first with 29,096 co-citations, followed by *Clinical Endocrinology* (8850), *European Journal of Endocrinology* (8635), *New England Journal of Medicine* (4345), and *The Lancet* (2316). Notably, *The Lancet* had the highest impact factor (IF = 168.9), followed by the *New England Journal of Medicine* (IF = 158.5). Among the top 10 co-cited journals, 7 (70%) were ranked as Q1, 2 (20%) as quartile 2, and 1 (10%) as quartile 3. Journals with a minimum of 500 co-citations were selected for the co-citation network (Fig. 4B). This visualization highlights the strong co-citation relationships of journals such as *The Journal of Immunology* with *Blood*, *PLOS ONE*, and *Frontiers in Immunology*.

The dual-map overlay (Fig. 4C) presents journal citation trajectories. Three major citation paths were identified. The orange path indicates that research published in *Molecular/Biology/Genetics* journals was primarily cited by *Molecular/Biology/Immunology* journals. The green paths show broader interdisciplinary connections, where studies published in *Molecular/Biology/Genetics/Health/Nursing/Medicine* journals were predominantly cited in *Medicine/Medical/Clinical* domains.

### 3.4. Analysis of authors and co-cited authors

**Figure 5.**
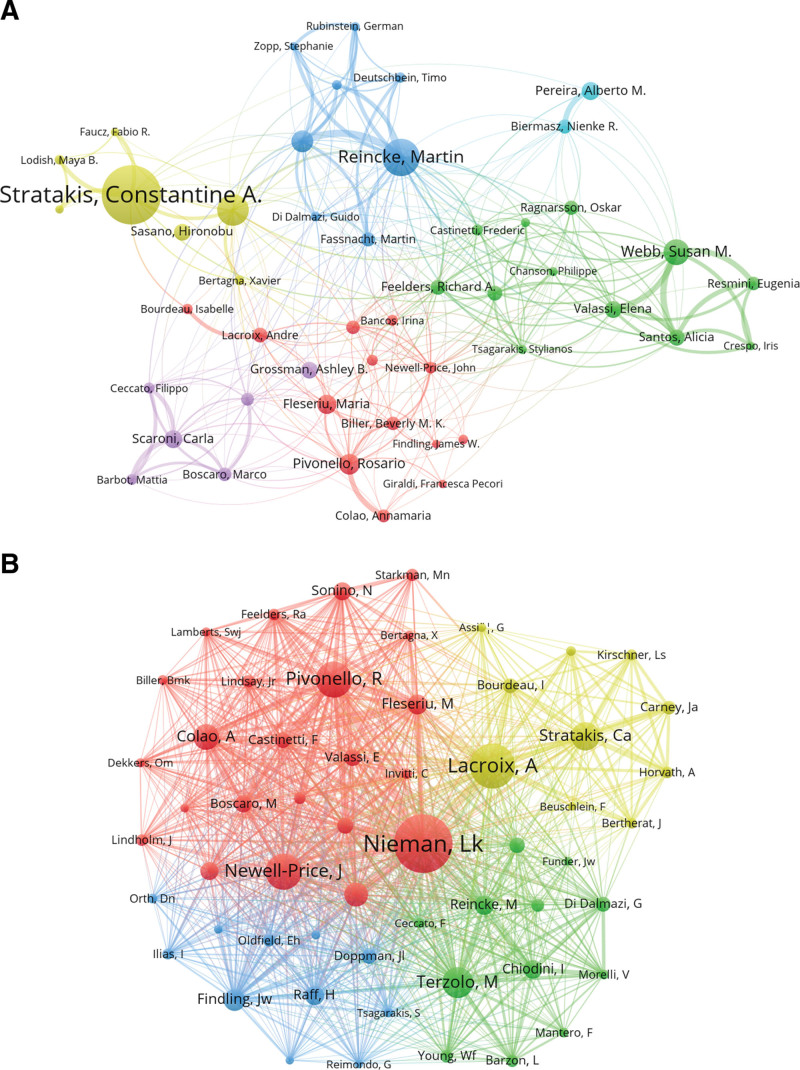
(A) Visualization of authors in CS research. (B) Co-cited network of authors in CS research. CS = Cushing syndrome.

A total of 19,621 authors contributed to CS research. The top 10 most profilic authors and their citation counts are listed in Table 3. A collaborative network was constructed for authors with at least 18 publications (Fig. 5A). Authors with the largest nodes, such as Constantine A. Stratakis, Martin Reincke, Jérôme Bertherat, Susan M. Webb, and Felix Beuschlein, had the highest publication output. Strong collaborative relationships were identified: Jerome Bertherat frequently coauthored with Constantine A. Stratakis; Susan M. Webb with Alicia Santos, Eugenia Resmini, and Elena Valassi; and Martin Reincke with Felix Beuschlein. These findings reflect a high degree of international cooperation and knowledge exchange among key contributors.

**Table 3:** Top 10 authors and co-cited authors on the research of CS.

Rank	Author	Documents	Co-cited authors	Citations
1	Constantine A Stratakis	124	Lynnette K Nieman	1552
2	Martin Reincke	79	André Lacroix	1197
3	Jérôme Bertherat	66	Rosario Pivonello	963
4	Susan M Webb	54	John Newell-Price	958
5	Felix Beuschlein	46	Massimo Terzolo	819
6	Rosario Pivonello	44	Constantine A Stratakis	758
7	Maria Fleseriu	41	Annamaria Colao	683
8	Alberto M Pereira	38	Giorgio Arnaldi	643
9	Carla Scaroni	38	James W Findling	584
10	Ashley B Grossman	36	Maria Fleseriu	543

CS = Cushing syndrome.

Table 3 also presents the top 10 co-cited authors. Lynnette K. Nieman was the most co-cited author (n = 1552), followed by André Lacroix (n = 1197) and Rosario Pivonello (n = 963). Authors who had a minimum of 220 co-citations were included in the author co-citation network (Fig. 5B), reflecting a dense and interconnected knowledge foundation shaped by repeated referencing of authoritative sources.

### 3.5. Analysis of co-cited references

**Figure 6.**
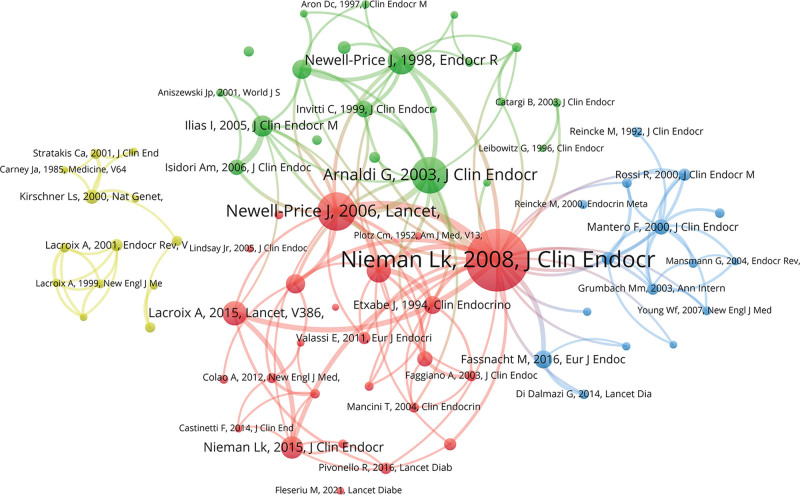
Visualization of co-cited references in CS research. CS = Cushing syndrome.

The top 10 co-cited references, listed in Table 4, each received at least 226 citations, with the most highly cited reference exceeding 700 citations. The most co-cited paper was authored by Nieman Lk in 2008, published in *The Journal of Clinical Endocrinology & Metabolism* (Vol. 93, p. 1526), which provided clinical guidelines for testing and diagnosing endogenous CS.^[9]^ The guideline recommends that patients undergo an initial high-sensitivity screening test. If both test results are abnormal, an etiological workup is necessary. If discordant results occur, periodic hypercortisolism should be considered. References with at least 90 co-citations were used to generate the co-citation network (Fig. 6A).

**Table 4:** Top 10 co-cited references on the research of CS.

Rank	Cited reference	Citations
1	Nieman Lk, 2008, J Clin Endocr Metab, V93, P1526, Doi 10.1210/Jc.2008-0125	742
2	Newell-Price J, 2006, Lancet, V367, P1605, Doi 10.1016/S0140-6736(06)68699-6	458
3	Arnaldi G, 2003, J Clin Endocr Metab, V88, P5593, Doi 10.1210/Jc.2003-030871	432
4	Newell-Price J, 1998, Endocr Rev, V19, P647, Doi 10.1210/Er.19.5.647	327
5	Lacroix A, 2015, Lancet, V386, P913, Doi 10.1016/S0140-6736(14)61375-1	288
6	Lindholm J, 2001, J Clin Endocr Metab, V86, P117, Doi 10.1210/Jc.86.1.117	288
7	Nieman Lk, 2015, J Clin Endocr Metab, V100, P2807, Doi 10.1210/Jc.2015-1818	266
8	Ilias I, 2005, J Clin Endocr Metab, V90, P4955, Doi 10.1210/Jc.2004-2527	249
9	Biller Bmk, 2008, J Clin Endocr Metab, V93, P2454, Doi 10.1210/Jc.2007-2734	232
10	Oldfield Eh, 1991, New Engl J Med, V325, P897, Doi 10.1056/Nejm199109263251301	226

CS = Cushing syndrome.

### 3.6. Analysis of references with citation bursts

**Figure 7.**
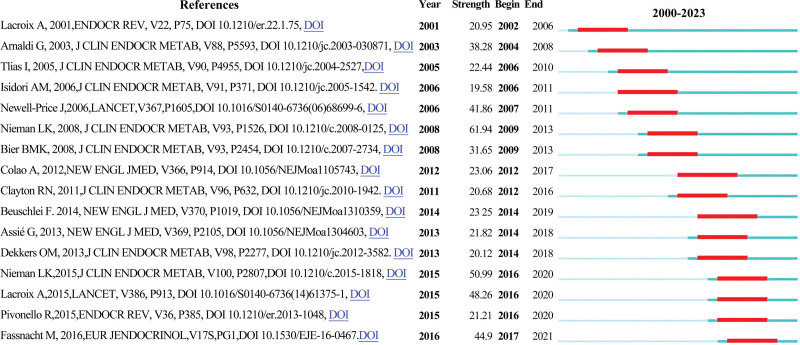
Top 10 references with the strongest citation bursts: red bars indicate peak citation years.

Citation bursts identify publications that have received a sharp increase in attention over a specific period, often indicating influential or emerging work. Sixteen references with significant citation bursts were detected using CiteSpace (Fig. 7A). Burst periods ranged from 2002 to 2021. The reference with the strongest citation burst (strength = 61.94) was “*The diagnosis of Cushing syndrome: an Endocrine Society Clinical Practice Guideline*,” authored by Lynnette K. Nieman et al, with the burst lasting from 2009 to 2013.^[9]^ The second strongest burst (strength = 50.99) occurred for “*Treatment of Cushing Syndrome: An Endocrine Society Clinical Practice Guideline*,” published in *The Lancet* by Lynnette K. Nieman et al, from 2016 to 2020.^[10]^ This reference provided detailed guidance on restoring normal cortisol levels through effective clinical intervention. Overall, the burst strengths ranged from 19.58 to 61.94, with durations of 5 to 6 years, indicating sustained influence over time.

### 3.7. Citation classics and non-classics

To distinguish foundational works from emerging studies, publications were classified as “citation classics” (≥500 citations) and “non-classics” (<500 citations), as presented in Table 5. Among the 16 citation classics, clinical practice guidelines (n = 5) and review articles (n = 9) dominated, highlighting their central role in conslidating knowledge and establishing standards of care. The remaining 2 publications were original research articles (n = 2).

**Table 5:** The citation classics on the research of CS.

Rank	Author (year)	Journal (IF)	DOI	Citations
1	Nieman (2008)	Journal of Clinical Endocrinology & Metabolism (5.80)	10.1210/jc.2008-0125	1611
2	Sapolsky (2000)	JAMA Psychiatry (25.80)	10.1001/archpsyc.57.10.925	1383
3	Newell-Price (2006)	Lancet (168.90)	10.1016/S0140-6736(06)68699-6	921
4	Arnaldi (2003)	Journal of Clinical Endocrinology & Metabolism (5.80)	10.1210/jc.2003-030871	910
5	Fassnacht (2016)	Journal of Neuroendocrinology (3.20)	10.1530/EJE-16-0467	909
6	Mantero (2000)	Journal of Clinical Endocrinology & Metabolism (5.80)	10.1210/jcem.85.2.6372	747
7	Speiser (2010)	Journal of Clinical Endocrinology & Metabolism (5.80)	10.1210/jc.2009-2631	706
8	Cohen (2015)	Nature Reviews Drug Discovery (120.10)	10.1038/nrd4467	669
9	Nieman (2015)	Journal of Clinical Endocrinology & Metabolism (5.80)	10.1210/jc.2015-1818	654
10	Liu (2013)	Allergy Asthma and Clinical Immunology (2.70)	10.1186/1710-1492-9-30	629
11	Biller (2008)	Journal of Clinical Endocrinology & Metabolism (5.80)	10.1210/jc.2007-2734	617
12	Allolio (2006)	Journal of Clinical Endocrinology & Metabolism (5.80)	10.1210/jc.2005-2639	536
13	Lindholm (2001)	Journal of Clinical Endocrinology & Metabolism (5.80)	10.1210/jcem.86.1.7093	531
14	Manolopoulos (2010)	International Journal of Obesity (4.90)	10.1038/ijo.2009.286	518
15	Lacroix (2015)	Lancet (168.90)	10.1016/S0140-6736(14)61375-1	515
16	Unger (2003)	Endocrinology (4.90)	10.1210/en.2003-0870	505

CS = Cushing syndrome.

The *Journal of Clinical Endocrinology & Metabolism* served as the principal publishing platform for these citation classics, accounting for 8 of the top 16 (50%). Notably, 12 of the top 16 classics (75%) appeared in journals classified as Q1 according to *Journal Citation Reports 2023*, reflecting their high impact and scholarly influence. Identifying citation classics provides insight into the field’s foundational literature and underlines key areas that continue to attract sustained research interest.

### 3.8. Hotspots and trends

**Figure 8.**
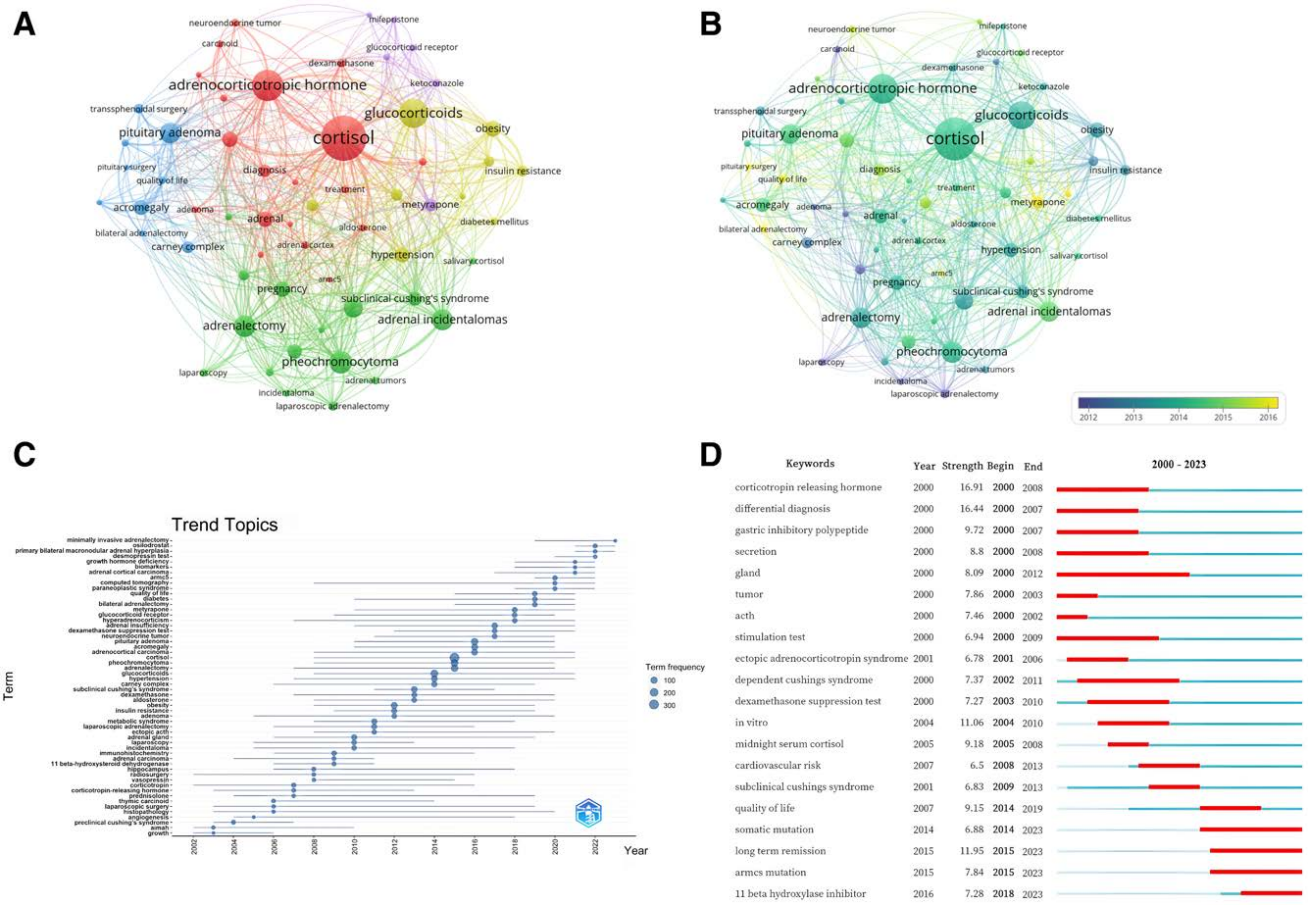
(A) Keyword co-occurrence network: node size indicates frequency; color indicates thematic clustering. (B) Overlay visualization of author keywords: color represents average year of appearance. (C) Trend topic analysis: node size indicates keyword frequency; line length denotes duration of prevalence. (D) Top 20 keywords with the strongest citation bursts: red lines indicate burst onset years.

Keywords serve as condensed representations of a study’s core concepts and thematic focus. To refine the keyword dataset, generic disease-related terms such as *Cushing syndrome*, *Cushing disease*, and *primary aldosteronism* were excluded. Additionally, nonspecific terms such as *society*, *European society*, *outcome, and masse* were removed. A total of 5610 author keywords were identified. Of these, 59 appeared more than 25 times (Fig. 8A). The most frequent keywords were “cortisol” (n = 308), “adrenocorticotropic hormone” (n = 196), and “glucocorticoids” (n = 180). Table 6 lists the 20 most frequently used keywords in CS research.

**Table 6:** Top 20 keywords on CS research.

Rank	Keywords	Occurrences	Rank	Keywords	Occurrences
1	Cortisol	308	11	Adrenal	84
2	Adrenocorticotropic hormone	196	12	Pregnancy	82
3	Glucocorticoids	180	13	Acromegaly	81
4	Pheochromocytoma	131	14	Hypertension	79
5	Adrenalectomy	127	15	Adrenocortical carcinoma	78
6	Adrenal incidentalomas	120	16	Subclinical Cushing syndrome	75
7	Pituitary adenoma	117	17	Metyrapone	68
8	Adrenal tumor	109	18	Diagnosis	63
9	Obesity	86	19	Carney complex	62
10	Pituitary	85	20	Osteoporosis	62

CS = Cushing syndrome.

Co-occurrence analysis grouped these keywords into 5 clusters. The red cluster relates to diagnostic studies and includes *cortisol*, *adrenocorticotropic hormone*, *adrenal*, *ectopic adrenocorticotropic hormone secretion*, and *dexamethasone*. The blue cluster centers on surgical approaches, including *bilateral adrenalectomy*, *pituitary surgery*, *transsphenoidal surgery*, and *quality of life*. The green cluster addresses etiology and includes *adrenal incidentalomas*, *adrenal tumor*, *adrenalectomy*, and *laparoscopic adrenalectomy*. The yellow cluster focuses on complications and comorbidities, with terms such as *glucocorticoids, obesity*, *diabetes mellitus*, *insulin resistance*, *a*nd *metabolic syndrome*. The purple cluster relates to pharmacotherapy and includes agents such as *mifepristone*, *metyrapone*, and *ketoconazole*. Overall, research in CS has concentrated on surgical treatment, pharmacologic management, and disease mechanisms.

Keyword overlay visualization (Fig. 8B) revealed that terms such as Armadillo repeat-containing protein 5 (ARMC5), *pituitary surgery*, and *metyrapone* have shown increasing frequency in recent years. Trend topic analysis (Fig. 8C) indicated that recent research (2022–2023) has focused on *minimally invasive adrenalectomy*, *osilodrostat*, *primary bilateral macronodular adrenal hyperplasia*, and *the desmopressin test*. Burst keyword analysis (Fig. 8D) identified *corticotropin-releasing hormone* and *differential diagnosis* as having the highest citation burst intensities. Currently, burst keywords include *11 beta-hydroxylase inhibitors*, *ARMC5 mutation*, and *somatic mutation*.

Although keywords such as *pituitary surgery*, *desmopressin test*, and *minimally invasive adrenalectomy* have relatively low frequencies, they may represent emerging directions for future research. Additionally, *primary bilateral macronodular adrenal hyperplasia* and *ARMC5 mutation* both associated with somatic mutation, along with therapeutic agents like *metyrapone*, *osilodrostat*, and *11 beta-hydroxylase inhibitors*, are likely to remain highly relevant research targets.

A comprehensive analysis of keyword co-occurrence, overlay, bursts, and trend reajectories suggests that *somatic mutation* and *11 beta-hydroxylase inhibitors* currently represent the most active research frontiers in the field of CS.

## 4. Discussion

### 4.1. General information

This study presents a comprehensive bibliometroc analysis of global research output on CS using data from the Web of Science Core Collection. A total of 4661 articles were retrieved, published across 1021 journals by 3972 institutions from 86 countries. Scientometric tools, including VOSviewer and CiteSpace, were applied to map the intellectual structure and emerging trends in the field.

The publication trend from January 2020 to October 2023 revealed a notable increase in the volume of CS-related articles, reflecting the field’s growing importance and research momentum. The Eunice Kennedy Shriver National Institute of Child Health and Human Development (USA) and the University of Padua (Italy) emerged as the leading institutions, contributing significantly to global CS research. Based on citation metrics and impact factor rankings, *The Journal of Clinical Endocrinology & Metabolism*, *European Journal of Endocrinology*, and *Clinical Endocrinology* were identified as the most influential publication venues, with a strong focus on clinical research. These journals maintain robust citation relationships with high-impact titles such as *New England Journal of Medicine* and *The Lancet*, both of which are ranked in the Q1 quartile of *Journal Citation Reports*. Notably, *The Lancet* has the highest impact factor (IF = 168.90). Collectively, these core journals play a central role in disseminating research related to diagnosis, treatment, and patient management in CS, thereby shaping the direction and quality of scholarly contributions in the field.

### 4.2. Current hotspot analysis and future directions

Analysis of highly cited references and author contributions demonstrates that CS research over the past 2 decades has emphasized the development of diagnostic criteria and clinical practice guidelines. Diagnostic strategies have included measurement of urinary free cortisol, late-night salivary cortisol, and dexamethasone suppression testing, along with more recent approaches such as hair cortisol. Treatment guidelines have focused on surgical interventions and adjunctive therapies, particularly steroidogenesis inhibitors.^[9–16]^ The prominence of clinical practice guidelines among the most cited references highlights the field’s commitment to translating evidence into standardized care pathways. Core research themes have included pathophysiological mechanisms (especially genetics), diagnostic accuracy, treatment efficacy, and ong-term outcomes including quality of life. Keywords and trend analyses (Fig. 8A–D) identified 2 major and evolving research frontiers.

#### 4.2.1. Somatic mutation

Somatic mutations associated with cortisol hypersecretion represent a critical area of investigation. Mutations in genes regulating the cyclic adenosine monophosphate/protein kinase A signaling pathway are well-established drivers of cortisol-producing adrenal adenomas and hyperplasia.^[17–19]^ These mutations result in constitutive activation of protein kinase A, promoting cortisol overproduction. Mutations in protein kinase cAMP-activated catalytic subunit alpha (PRKACA) are especially prevalent in unilateral adenomas, while protein kinase cAMP-activated catalytic subunit alpha copy number gains are associated with bilateral adrenal hyperplasia.^[19]^ Mutations in catenin beta 1 have also been implicated in cortisol-producing adenomas.^[19,20]^

Inactivating mutations in the tumor suppressor gene ARMC5 are a leading cause of primary bilateral macronodular adrenal hyperplasia and subsequent CS. In corticotroph adenomas causing Cushing’ disease, somatic mutations in genes encoding deubiquitinases particularly Ubiquitin-Specific Protease 8, and less frequently, ubiquitin-specific protease 48 have been discovered.^[21]^ These mutations enhance epidermal growth factor receptor signaling and increase transcription of pro-opiomelanocortin and secretion of adrenocorticotropic hormone. Mutations in Ubiquitin-Specific Protease 8 are associated with specific tumor characteristics and may influence responses to diagnostic testing.^[22]^

Ongoing research continues to investigate the functional consequences of these mutations, including effects on apoptosis, receptor expression, and cell cycle regulation.^[23–26]^ Overall, the characterization of somatic mutations contributes to a deeper understanding of CS pathogenesis and may support the development of targeted therapies.

#### 4.2.2. 11β-Hydroxylase inhibitors

Pharmacological inhibition of 11β-hydroxylase, a key enzyme in cortisol biosynthesis, is an important therapeutic approach for patients who are not surgical candidates or who have recurrent disease.^[27]^ Osilodrostat and metyrapone are the primary agents in this class. Clinical studies have demonstrated the efficacy of osilodrostat in rapidly reducing urinary free cortisol levels, leading to its regulatory approval.^[28]^ Ongoing research focuses on refining dosage strategies (e.g., initial dosing of 10 mg twice daily), managing drug interactions (osiolodrostat inhibits cytochrome P450 3A4, cytochrome P450 1A2, cytochrome P450 2C19), and characterizing adverse events such as adrenal insufficiency, hypokalemia, hypertension, and androgen excess.^[29–31]^

Comparative data suggest differences in the profiles of enzyme inhibition between osilodrostat and metyrapone, with potential advantages of osilodrostat in long-term blood pressure control.^[32]^ Metyrapone remains a key therapeutic option, particularly in specific populations such as pregnant women and pediatric patients^.[33,34]^ Combination therapies (e.g., osilodrostat with ketoconazole) are also under investigation for treatment-refractory CS.^[35]^ Future research is expected to optimize the clinical use of existing inhibitors, mitigate side effects, explore novel combinations, and develop new agents with improved selectivity and safety profiles.

## 5. Strengths and limitations

This study offers several contributions. To our knowledge, it is the first comprehensive bibliometric analysis of CS research, providing a valuable reference framework for researchers and clinicians in the field. The complementary use of 3 bibliometric tools, named VOSviewer, CiteSpace, and bibliometrix, enhanced the methodological rigor of the analysis. The combined application of these platforms, each widely adopted in bibliometric research, increases the reliability and objectivity of the findings. Moreover, compared to conventional narrative reviews, bibliometric methods allow for a more systematic and quantitative assessment of publication trends, collaboration patterns, and research frontiers, thereby offering a deeper and more integrative understanding of the field’s evolution.

Nonetheless, this study has limitations. The analysis was restricted to publications indexed in the Web of Science Core Collection, which may have excluded relevant articles from other databases such as Scopus or PubMed. Additionally, citation metrics are inherently time-dependent. Recent high-quality studies may not yet have accumulated citations, leading to possible underrepresentation of emerging contributions. Finally, keyword normalization remains challenging, and variations in terminology may have impacted the clustering of related topics.

## 6. Conclusion

This study provides a comprehensive bibliometric assessment of CS research from 2000 to 2023, synthesizing the knowledge base, research hotspots, and emerging trends that have shaped the field. Through the integration of multiple visualization tools and robust analytic methods, we identified the most influential countries and institutions, core journals, prolific authors, collaborative research networks, highly cited references, and high-frequency keywords.

The results highlight the prominence of clinical research and guideline development over the past 2 decades, with growing attention to genetic mechanisms and targeted therapies. Two key research trends, somatic mutations and 11 beta-hydroxylase inhibitors, are expected to drive future advances in CS diagnosis and treatment. By mapping trajectory of CS authorship, this study serves as a foundational reference for future research planning, interdisciplinary collaboration, and policy development in the management of CS.

## Acknowledgments

The authors express their sincere gratitude to all individuals who contributed to the completion of this study.

## Author contributions

**Conceptualization:** Xiao Lu, Quanyuan Huang, Yufang Yang.

**Data curation:** Xiao Lu, Quanyuan Huang, Mingyu Huang.

**Funding acquisition:** Xiao Lu, Quanyuan Huang.

**Investigation:** Yufang Yang, Dongxiao Chen, Fuli Qin.

**Methodology:** Quanyuan Huang.

**Software:** Quanyuan Huang, Dongxiao Chen, Fuli Qin.

**Validation:** Yufang Yang, Dongxiao Chen, Fuli Qin.

**Visualization:** Xiao Lu, Mingyu Huang.

**Writing – original draft:** Xiao Lu, Mingyu Huang.

**Writing – review & editing:** Xiao Lu, Quanyuan Huang, Yufang Yang.
